# Gender differences in a resources-demands model in the general population

**DOI:** 10.1186/1471-2458-14-902

**Published:** 2014-09-01

**Authors:** Rüya-Daniela Kocalevent, Burghard F Klapp, Cornelia Albani, Elmar Brähler

**Affiliations:** Institute and Policlinic for Medical Psychology, University Medical Center Hamburg-Eppendorf, Martinistr 52 (Haus W26), Hamburg, 20246 Germany; Department of Psychosomatis, Charité University Medicine Berlin, Charitéplatz 1, Berlin, 10117 Germany; Department of Medical Psychology and Medical Sociology, University of Leipzig, Germany, Ph.-Rosenthal-Str. 55, Leipzig, 04103 Germany; Department of Psychosomatic Medicine and Psychotherapy, Universal Medical Center Mainz, Mainz, Rhinland-Palatinate, Germany

**Keywords:** Gender, Resources, Chronic work stress, Exhaustion, General population

## Abstract

**Background:**

The population-based study examined postulated effects, derived from a resources-demands-model about gender-related aspects of self-efficacy, optimism, chronic stress, and exhaustion.

**Methods:**

Data acquisition was carried out by a market research institute with a multi-topic questionnaire in the general population (N = 2,552). Instruments administered were the Questionnaire for Self-Efficacy and Optimism, the Trier Inventory for Chronic Stress, and the Chalder-Fatigue-Scale. Households and target persons were selected randomly. The analyses focused on structural equation modeling.

**Results:**

There were significant differences in structural relations among the *resource* paths. In particular, significant gender differences were found with respect to *self-efficacy*, and among the *exhaustion* paths, namely in the *mental dimension* of *exhaustion*. The observed measures of *chronic stress* were found to be operating equivalently for both genders. Results suggest that resources play an important role in the understanding of how chronic stress is preceded and may lead to exhaustion in both genders.

**Conclusion:**

Personal resources seem to be more expressed by men than by woman, for whom the relation of resources to health is of greater importance than for men.

## Background

The recent German Health Interview and Examination Survey for Adults reports high levels of chronic stress significantly more often for women (13.9%) than for men (8.2%), yet not reporting on effect sizes [1]. A preliminary study in the general population estimated the prevalence rate for elevated chronic stress to be 17.7% for men and 20.5% for women, but differences were not significant (p = 0.08) [2]. Furthermore the frequency with which moderate to high acute stress is experienced in the general population was documented to be 17.9% for men and 21.4% for women, showing also rather small effect sizes (Cohen’s d = 0.13) [3].

An increase in stress and exhaustion can be interpreted as the answer to prolonged emotional and/or interpersonal stressors experienced in the work place [4]. A diverse range of occupational groups are affected and not, as previously assumed, only the so called ‘caring professions’ i.e. nurses and doctors or teachers and social workers [5–9]. Those people practising the same tasks for a long time seem to be especially at risk (>16 years) [10], as are those with unhealthy lifestyles (e.g. substance abuse), which often represents a dysfunctional strategy for coping with stress [11]. Limits to personal capacities in such circumstances are often recognised too late, and the potential of preventative psychotherapeutic measures, such as EAP (Employee Assistance Program) not fully applied [12]. Not only the employed are affected; those studying who are unable to cope with performance pressures [13], those who are newly employed, as well as the unemployed complain of experiencing increased stress and show signs and symptoms of subjectively experienced emotional and physical exhaustion [14]. Studies investigating gender differences in occupational stress have produced contradictory results, with some to indicate no differences, and some suggesting that either men or women experience more psychological stress. In the *Job stress, Absenteeism and Coronary heart disease in Europe (JACE) study*, men perceived marginally less job-demand as compared to women. Differences were larger for control: men appeared to perceive more control at work than did women [15]. Other results did not indicate significant gender differences, when marital status, age, and education were introduced into the equation [16]. Besides these work related stressors the so-called nonwork stress – defined as “hassels” (health, family, and social, environmental, financial) - seems to be of greater valence for women than for men [17]. A meta- analysis on gender differences in burnout yielded rather small effect sizes [18]. Yet, female employees were more likely to experience burnout than male employees, revealing that women were more emotionally exhausted than men, while men were more depersonalized than women.

Resources can directly affect mental- and physical exhaustion or well-being, by positively influencing, prevailing events between stressor and stress reaction in both genders [19, 20]. Resources attributed to an individual (personal resources) are differentiated from those which form parts of the life environment (external resources). The meaning of personal resources encompasses the availability of psychological and physical attributes (i.e. good physiological condition, dispositional optimism, communicative skills or general self-efficacy) [21].

General self-efficacy is hereby a resource that may buffer the way in which burdening and challenging chronic stressors are dealt with; this means, the extent to which a person is convinced that she can perform appropriately in a specific situation. Self-efficacy further influences self-perception, motivation and performance in a variety of ways, and is regarded as a good predictor for preventative health behaviour [22]. Earlier studies suggest that the construct is universal, showing slightly higher mean scores for men in the majority of cultures worldwide [23].

In general, studies investigating gender differences in stress responses indicate that while women show greater psychological reactivity to stress (see [24] for a review), men show greater physiological reactivity to stress [25].

In a previous study on the associations of resources, chronic activated distress, and exhaustion in general, Kocalevent et al. [2] could demonstrate the influence of chronic stress on exhaustion diminishes when the direct influence of resources on exhaustion is taken into account. The analyses focused on structural equation modeling and hypotheses were derived from a resources-demands model of health [26]. This resources-demands model records chronic stress as subjective perceived stress. Stress factors and subjective perceived stress should be unspecific and at the same time interpretable to a variety of real-life situations (e.g., “you feel under pressure from deadlines”), and (c) the perceived stress should be recorded independently of the stage in the coping process at which the subject might currently be, other than resources-demands models focusing only on employees [27].

This resources oriented model is based on the supposition that people’s estimations of their resources determine the experienced amount of exhaustion; and that what is stressful to them is the potential or actual loss of these resources [2, 26]. Resources then, are the unit necessary for understanding chronic stress. However, the mechanisms underlying gender-related chronic stress processes in a resources-demands model remain to be explicated. Special issues under investigation here are: Taking chronic stress into account, we focus on the way, in which gender differences in the general population are linked to exhaustion and personal resources, namely self-efficacy and dispositional optimism. First, if we include measures of resources, in addition to chronic stress - do we still observe gender differences in consequences of health, namely exhaustion? Second, does exposure to chronic work stressors explain gender differences in exhaustion? Third, which sociodemographic variables should be taken into account?

## Methods

### Study sample

A nationwide survey, representative of the German general population, was conducted with the assistance of an institute specialized for demographic research (USUMA, Berlin) according to the German law of data protection (§30a BDSG) and with written consent and in accordance with the guidelines in the Declaration of Helsinki, approved by the ethics committee of the University of Leipzig. All households and the target population (n = 3125) were randomly selected. The response rate was 81% (n = 2552). As part of the survey, participants were interviewed face-to-face at their home and shown the relevant questionnaire during the interview. Age, gender, and educational level were the major criteria for representativeness according to the register of the Federal Elections. Although the statistics represented the German population (age 16 and above) living in private households, only data relating to persons aged 18 and older was analysed. Descriptive parameters of the samples are shown in Table 1. Study participants were asked to (a) provide personal information (socio-demographic variables), and (b) to complete a comprehensive questionnaire which included the Trier Inventory of Chronic Stress (TICS) [28], the Chalder-Fatigue Scale (CSF) [29] and a sub questionnaire on self-esteem and optimism (SWOP) [30] to capture personal resources.

**Table 1:** **Study sample (N = 2552)**

Sociodemographic variables	Men (n = 1,206) n (%)	Women (n = 1,312) n (%)	Men n (%) chronic stress high ^a^	Significance men chronic stress high	Women n (%) chronic stress high	Significance women chronic stress high
**Age**				χ^2^(4) = 21.33; p < 0.01		χ^2^(4) = 21.33; p < 0.01
16-40 years	444 (36.8)	516 (37.9)	92 (20.7)		124 (24.4)	
41-60 years	375 (31.1)	426 (31.6)	76 (20.3)		86 (20.1)	
61-95 years	361 (29.9)	376 (27.9)	41 (11.4)		56 (15.1)	
**Cohabitation**				χ^2^(2) = 5.69; p = 0.06		χ^2^(2) = 0.93; p = 0.62
Yes	750 (62.2)	745 (55.3)	120 (16.1)		145 (19.6)	
No	456 (37.8)	601 (44.7)	93 (20.5)		129 (21.6)	
**Primary occupation**				χ^2^(16) = 47.06; p < 0.01		χ^2^(16) = 18.77; p = 0.28
Full-time (≥35 h/week)	606 (50.2)	327 (24.3)	118 (19.6)		86 (26.3)	
Part-time (15–34 h/Woche)	13 (1.1)	185 (13.7)	7 (53.9)		40 (21.7)	
Part-time (<15 h/Woche)	4 (0.3)	47 (3.5)	1 (25.0)		8 (17.1)	
Military service/maternity leave	7 (0.6)	30 (2.2)	2 (28.6)		6 (20.0)	
Retired	379 (31.4)	377 (28.0)	42 (11.1)		59 (15.9)	
Professional education	25 (2.1)	12 (0.9)	4 (16.0)		2 (18.2)	
Students	81 (6.7)	89 (6.6)	16 (19.8)		21 (23.6)	
**Unemployment**				χ^2^(2) = 18.01; p < 0.01		χ^2^(2) = 0.32; p = 0.85
No	1121 (93)	1253 (93.1)	192 (17.2)		253 (20.4)	
Yes	85 (7.0)	93 (6.9)	21 (24.7)		21 (22.6)	

^a^chronic stress high = > mean + ≥1 standard deviation.

### Instruments

#### Chronic Stress: TICS – Trier Inventory to Chronic Stress

The Trier Inventory to Chronic Stress (TICS) is a standardised questionnaire comprising 57 items for the differentiated diagnostic of various facets of chronic stress [28, 31]. In answering the TICS, study participants provided information about how often they experienced a particular situation in the previous three months. The TICS questionnaire comprises ten scales. Three deal with stress associated with *occupational stress* and *social overload* and *pressure to succeed*; five scales deal with stress associated with *work dissatisfaction, work overload*, *lack of social recognition/support, social tension* and *social isolation*, which is directly linked to a lack of fulfilment needs (Cronbach’s α = .84 to .91). Furthermore, the TICS instrument includes a scale for *chronic worrying* as well as a 12-item screening scale (which measures the total chronic stress experienced). All scales satisfy the ordinal Rasch-model (*RR* = .78 to .89). The cut-off point for the screening scale of *chronic stress* was calculated by analogy to a previous stress study [2]: ‘chronic stress high’ group was defined as: mean + ≥1 standard deviation.

#### Mental and physical exhaustion: CFS-Chalder Fatigue Scale

The Chalder-Fatique scale has been developed to capture the extent of mental and physical fatigue [29]. The CFS comprises 11 items and is reliable (physical fatigue: α = .85; mental fatigue α = .82; total scale: α = .89), valid and culturally sensitive [32, 33]. Fatigue is defined as a continuum – not categorically. The possible response options to the questions asked were: 0 = ‘lesser than usual’, 1 = not more than usual’ and 2=‘more than usual’. Symptoms which are associated with a Chronic Fatigue Syndrome were not in focus.

#### Resources: SWOP – Questionnaire on Self-efficacy and Optimism

The questionnaire on self-efficacy and optimism is considered as valid and reliable (Cronbach’s α = .79) [29]. The questionnaire covers 9 items with four possible response categories: ‘not true’, ‘unlikely true’, ‘most likely true’ and ‘true’. Expectations of self-efficacy are defined as a source of generalised problem-solving, which is reflective of an individual’s sense of his own competency and ability. Optimism is defined as a person’s ability to channel attitude in such a way that it will have an advantageous effect in dealing with change on various levels [34].

### Data analysis

Structural equation modeling was used to enable a clearer conceptualization of the resources-demands-model under study. We used the following fit-indices, according to Tanaka [35]: comparative fit-index, CFI-fit-index (≥0,95 acceptable, ≥ 0,97 good) and the Tucker Lewis Index, TLI-fit-index (≥0,95 acceptable, ≥ 0,97 good) [35]. For the determination of differences between the groups (see Table 1) as well as for the regression analysis, the chronic stress screening scale (TICS) was used. All TICS scales were also included into the structural equation model. Additional analyses were conducted to test the invariance of the model across both gender using multi-group CFA. This is an important statistical condition before the means of different subgroups can be compared with each other. The measurement invariance was tested using the configural, combined model (no constraints), followed by a metric invariant model (with equal item loadings, that is, the paths and covariances were constrained to be equal) [36]. Invariance tests have proven themselves as a necessary step in group analyses (e.g. gender, age, cross-culture).

All analyses were carried out with the statistics software for social sciences (SPSS) version 18.0 and AMOS. The statistical significance was determined at the α = 0.05 level.

## Results

### Sociodemographic variables

The gender ratio is balanced (female: *n* = 1346, 52.7%; male: *n* = 1206, 47.2%). Table 1 shows significant differences in chronic stress for age in both genders, and for primary occupation and unemployment for men only. Women in the 16–40 year range and men employed part-time are particularly affected. Rates of chronic stress are least expressed by retired men, aged > 61 years. Employed persons report less chronic stress than unemployed.

### Prevalence

19.1% of the participants reported high stress levels, with a gender distribution of 17.7% for men, and 20.5% for women. The reported gender differences in frequencies did not reach a level of significance (χ^2^(df = 1) = 3.10; p = 0.08).

Table 2 shows the odds ratios. The category with the lowest risk factor, defined as lowest prevalence rate, was the selected reference category [37]. The slightly higher risk among women was again not significant (p=0.07). Relevant chronic activated stress had a higher probability in the categories age (for both genders), cohabitation and socio-economic factors (both for men only). The highest risk for an elevated stress level was found in the lower social strata for men, according to the social-layer index by Winkler and Stolzenberg [38].

**Table 2:** **Odds ratios und prevalence rates for chronic stress**

Sociodemographic variables	Chronic stress ^b^ (n = 487; 19.1%)		Odds ratio ^c^ (95% CI)	
	Men	Women	Men	Women
**Age**				
18-40 years	92 (20.7%)	124 (24.4%)	2.03** (1.36-3.03)	1.81** (1.28-2.58)
41-60 years	76 (20.3%)	86 (20.1%)	1.98** (1.31-2.98)	1.43 (0.98-2.07)
61-95 years	41 (11.4%)	56 (15.1%)	1	1
**Cohabitation**				
Yes	120 (16.1%)	145 (19.6%)	1	1
No	93 (20.5%)	129 (21.6%)	1.35* (1.01-1.83)	1.13 (0.87-1.48)
**Socio-economic-status** ^**e**^				
Low	112 (21.4%)	133 (22.0%)	2.11* (1.17-3.80)	1.13 (0.62-2.05)
Medium	54 (13.8%)	96 (18.7%)	1.24 (0.66-2.31)	0.92 (0.51-1.69)
High	14 (11.5%)	15 (20.0%)	1	1

^b^logistic regression.

*p < 0.01.

**p < 0.05.

^e^Winkler und Stolzenberg (1999) [38].

### Gender differences

**Figure 1:**
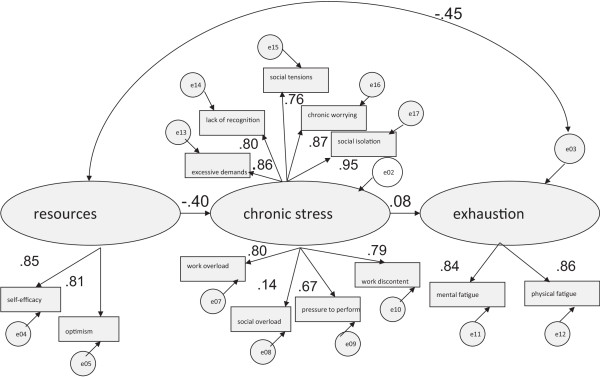
**Resources-demands-model: for women only (n = 1,346).**

**Figure 2:**
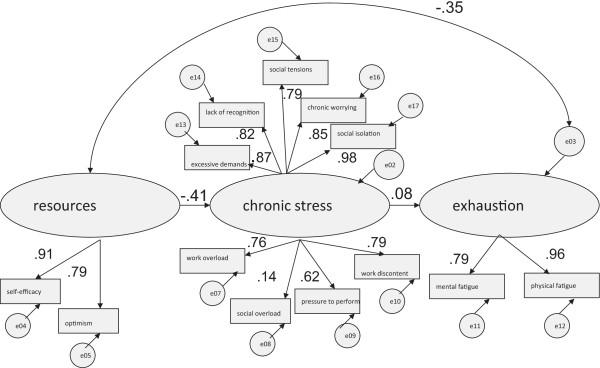
**Resources-demands-model: for men only (n = 1,206).**

Based on the theoretical and empirical framework [2], Figures 1 and 2 show the results for men and women with respect to the influences of resources and chronic stress on exhaustion. Using the measurements recommended by Tanaka [35] the depicted structural equation models in Figures 1 and 2, indicate satisfactory model fits (for men: TLI = 0.96; Comparative Fit Index, CFI = 0.96; for women: TLI = 0.96; CFI = 0.97).

For women *exhaustion* (.45), was predicted rather more by *resources* than it was for men (.35). Regarding *resources*, the path coefficient of *self-efficacy* is higher for men (.91) than for women (.85). Within the dimension of *chronic stress* (TICS-scales), path coefficients do not apparently differ. In terms of *exhaustion*, men show lower path coefficients in *mental fatigue* and a higher value in *physical fatigue* in contrast to women. Statistical significances of these findings are evaluated in the following section by testing for invariance of the model across gender (see Table 3).

**Table 3:** **Goodness-of-fit statistics for tests of invariance, for different gender groups of the hypothesized model: a summary**

	Model description	*χ* ^*2*^	*df*	Δ*χ* ^*2*^	Δ*df*	*Statistical significance*
	Baseline (male)	2841.7	74	-	-	*-*
	Baseline (female)	3029.5	74	-	-	*-*
1	Combined baseline model^1^	**5871.2**	**148**	-	-	*-*
2	Factor loadings, variances, and covariances constrained equal	5905.7	162	34.5	14	*p<0.01*
3	Factor loadings constrained equal	5903.1	159	31.9	11	*p<0.01*
4	Model 3 with factor loadings on *resources* constrained equal	5874.5	150	3.3	2	*n.s.*
5	Variances constrained equal	5871.4	150	0.2	2	*n.s.*
6	Covariances constrained equal	5873.8	149	2.6	1	*n.s.*
7	Covariances, resources, stress, exhaustion constrained equal	5874.7	151	2.9	3	*n.s.*
8	Covariances, resources, stress, exhaustion, self-efficacy constrained equal	5880.2	152	9	4	*p<0.05*
9	Model 8 with covariances, resources, stress, exhaustion, mental fatigue constrained equal	5884.5	153	13.3	5	*p<0.05*

^1^The combined baseline model provides the cut-point against which all subsequent models will be compared.

As shown in Table 3 the χ^2^-value for the combined baseline models (groups: males and females) was 5871.2 with 148 degrees of freedom. This χ^2^-value provides the cut-point against which all subsequent models will be compared in the series of tests to determine evidence of gender invariance. As shown in the upper part of Table 3, comparison of the combined baseline models (with equality constraints) with the original unconstrained model yields a χ^2^-difference value of 34.5 with 14 degrees of freedom, which is statistically significant at the .001 probability level. Presented with these findings, our next task was to analyse separately for factor loadings, variances, and covariances to locate nonequivalent parameters in the model.

Overall, the results summarized in Table 3 reveal *chronic stress* structure to be well described comprising the facets of *occupational stress* and *social overload* and *pressure to succeed*, as well as *work dissatisfaction, work overload*, *lack of social recognition/support, social tension* up to *social isolation* for both males and females.

However, although the observed measures of the TICS were found to be operating equivalently for both genders there were some differences in structural relations among the *resource* paths. In particular, significant gender differences were found with respect to *self-efficacy*, and among the *exhaustion* paths, namely in the *mental dimension* of *exhaustion*. The higher path coefficient of *self-efficacy* for men was therefore statistically significant, as well as the higher path coefficient of the *mental dimension* of *exhaustion* for women.

## Discussion

Despite the central role of resources in contemporary theories of the stress process, little is known about the determinants of resource change. Moreover, most stress studies have not incorporated the influence of resources on health outcomes. One exception to resource-static models is Hobfoll’s theory on conservation of resources (1989) [39]. According to this theory, resource loss is the primary operating mechanism driving stress reactions, as for example experience of exhaustion. In a 10-year longitudinal study, results showed that change in personal and social resources entirely mediated the effect of stressful life events [40]. Another resources-demands model focusing on employees also derived evidence for resources within stress processing on health outcomes [27].

The present study included measures of resources in addition to chronic stress and observed the effects on exhaustion. The evidence for a resources-demands-model tested within a structural regression model was confirmed by empirical data to be an accurate operationalization for both genders, yet with differences in *resources*, namely *self-efficacy*, and *exhaustion*, *mental fatigue* respectively. According to a resource-demand-model, men reported higher levels of resources than women. This disposition, or the lack of it, affects the experienced amount of exhaustion and possible consequences for health, as for example health-related quality of life, which is reported to be better in all areas by men, compared to women [41]. Furthermore the present study found higher scores for chronic stress in women than in men, yet the differences were not significant. This lack of significant results or small effect sizes concerning gender effects for chronic stress, corresponds with preliminary work on acute stress perception in the general population [3, 30, 42–44]. Other results from a sample of 2775 professionals suggested that women experience higher levels of occupational stress than men [16]. Nevertheless, when marital status, age and education were introduced in the equation, no significant gender differences were identified.

Further results from our cross-sectional sample suggest that chronic stress seems to rise and fall over the lifetime in a consistent way for both genders. The peak in the range from 16–40 years – early adulthood – might be due to the challenges in the fields of work and family management in this period of life [1]. After considering working conditions, engagement, and work-family conflicts – gender explains second best the variance of perceived job stress [45].

In addition, unemployment accounted for significant higher chronic stress in men than for women in the present study, as well as a low socio-economic status. The socioeconomic status was recorded via a multidimensional index which included information on education attainment, occupational status and household income [46]. Recent results from the general population reassure once again that persons with a low socioeconomic status have a self-rated health status which is worse than that of persons with a medium or high socioeconomic status [47]. Interestingly, although men reported significant higher chronic stress [47], a poorer general health status in low socio-economic status is reported more often by women. Another study [48] on gender differences, focusing on the onset of depression following a stressful life event in couples, revealed that rates of stress and depression in the general population can be a consequence of role differences: Women were found to have a greater risk of a depressive episode following a stressful life event than men, if those couples had a clear gender difference in associated roles, so that women held themselves more responsible for events associated with children and housing, and on the other hand enabled men to distance themselves from these issues. These results correspond to analysis of population-based samples between the 1960’s and 1980’s [49] and can support the assumption that role differences might have decreased over the past decades, at least in the industrial countries, and explain the fading effects on gender differences in stress scoring. Yet, differences in self-rated health status and quality of life remain to be in disadvantage for women associated with a less favourable socio economic status [41, 50].

The present study showed a strong link between negative signs for health, in terms of exhaustion, and aspects of personal resources. Moreover, the data from a representative population suggest that the relationship between health and stress is dependent more on age than on gender. A potential limitation of this general population study is that it is a cross-sectional study which would does not allow for interpretations of causality or possible mediator effects.

## Conclusion

Taken together, our results suggest that resources play an important role in the understanding of how chronic stress is proceeded and may or may not lead to exhaustion in both genders. Yet, personal resources seem to be more experienced by men than by woman, for whom the relation of resources to health is of greater importance than for men.

## References

[1] Hapke U, Maske UE, Scheidt-Nave C, Bode L, Schlack R, Busch MA (2013). [Chronic stress among adults in Germany : Results of the German Health Interview and Examination Survey for Adults (DEGS1)]. Bundesgesundheitsbla.

[2] Kocalevent RD, Klapp BF, Albani C, Brahler E (2013). [Associations of resources factors, chronic activated distress, and fatigue in the German general population]. Psychother Psychosom Med Psychol.

[3] Kocalevent RD, Hinz A, Brahler E, Klapp BF (2011). [Regional and individual factors of stress experience in Germany: results of a representative survey with the perceived stress questionnaire (PSQ)]. Gesundheitswesen.

[4] Rixgens P, Badura B (2012). Zur Organisationsdiagnose psychischen Befindens in der Arbeitswelt. Bundesgesundheitsbla.

[5] Leiter MP, Frank E, Matheson TJ (2009). Demands, values, and burnout: relevance for physicians. Can Fam Physician.

[6] Leiter MP, Maslach C (2009). Nurse turnover: the mediating role of burnout. J Nurs Manag.

[7] Maslach C, Schaufeli WB, Leiter MP (2001). Job burnout. Annu Rev Psychol.

[8] van Dick R, Wagner U (2001). Stress and strain in teaching: a structural equation approach. Br J Educ Psychol.

[9] Fothergill A, Edwards D, Burnard P (2004). Stress, burnout, coping and stress management in psychiatrists: findings from a systematic review. Int J Soc Psychiatry.

[10] Ahola K, Honkonen T, Isometsa E, Kalimo R, Nykyri E, Koskinen S, Aromaa A, Lonnqvist J (2006). Burnout in the general population. Results from the Finnish Health 2000 Study. Soc Psychiatry Psychiatr Epidemiol.

[11] Ahola K, Pulkki-Raback L, Kouvonen A, Rossi H, Aromaa A, Lonnqvist J (2012). Burnout and behavior-related health risk factors: results from the population-based Finnish Health 2000 study. J Occup Environ Med.

[12] Burnus M, Benner V, Kirchner D, Drabik A, Stock S (2012). [Comparison of two access portals of an employee assistance program at an insurance corporation targeted to reduce stress levels of employees]. Versicherungsmedizin.

[13] Gumz A, Brahler E, Erices R (2012). Burnout und Arbeitsstörungen bei Studenten. Psychother Psychosom Med Psychol.

[14] Neuberger O (1994). Personalentwicklung.

[15] de Smet P, Sans S, Dramaix M, Boulenguez C, de Backer G, Ferrario M, Cesana G, Houtman I, Isacsson SO, Kittel F, Ostergren PO, Peres I, Pelfrene E, Romon M, Rosengren A, Wilhelmsen L, Kornitzer M (2005). Gender and regional differences in perceived job stress across Europe. Eur J Public Health.

[16] Michael G, Anastasios S, Helen K, Catherine K, Christine K (2009). Gender differences in experiencing occupational stress: The role of age, education and marital status. Stress and Health.

[17] Hogan JM, Carlson JG, Dua J (2002). Stressors and stress reactions among university personnel. Int J Stress Manag.

[18] Purvanova RK, Muros JP (2010). Gender differences in burnout: a meta-analysis. J Vocat Behav.

[19] Bovier PA, Chamot E, Perneger TV (2004). Perceived stress, internal resources, and social support as determinants of mental health among young adults. Qual Life Res.

[20] Segerstrom SC, Taylor SE, Kemeny ME, Fahey JL (1998). Optimism is associated with mood, coping, and immune change in response to stress. J Pers Soc Psychol.

[21] Lazarus RS (1989). Psychological stress in the workplace. J UOEH.

[22] Luszczynska A, Scholz U, Schwarzer R (2005). The general self-efficacy scale: multicultural validation studies. J Psychol.

[23] Schwarzer R (1999). Self-regulatory Processes in the Adoption and Maintenance of Health Behaviors. J Health Psychol.

[24] Kudielka BM, Hellhammer J, Hellhammer DH, Wolf OT, Pirke KM, Varadi E, Pilz J, Kirschbaum C (1998). Sex differences in endocrine and psychological responses to psychosocial stress in healthy elderly subjects and the impact of a 2-week dehydroepiandrosterone treatment. J Clin Endocrinol Metab.

[25] Flinn MV, Quinlan RJ, Decker SA, Turner MT, England BG (1996). Male–female differences in effects of parental abscence on glucocorticoid stress response. Hum Nat.

[26] Becker P, Bös K, Woll A (1994). Ein Anfoderungs-Ressourcen-Modell der körperlichen Gesundheit: Pfadanalytische Überprüfungen der latenten Variablen. Z Gesundh.

[27] Bakker AB, Demerouti E (2006). The Job Demands-Resources model: state of the art. J Managr Physiol.

[28] Schulz P, Schlotz W, Becker P (2004). TICS - Trierer Inventar zum chronischen Stress.

[29] Chalder T, Berelowitz G, Pawlikowska T, Watts L, Wessely S, Wright D, Wallace EP (1993). Development of a fatigue scale. J Psychosom Res.

[30] Scholler G, Fliege H, Klapp BF (1999). Fragebogen zu Selbstwirksamkeit, Optimismus und Pessimismus. Psychother Psychosom Med Psychol.

[31] Becker J, Fliege H, Kocalevent RD, Bjorner JB, Rose M, Walter OB, Klapp BF (2008). Functioning and validity of a Computerized Adaptive Test to measure anxiety (A-CAT). Depress Anxiety.

[32] Wong WS, Fielding R (2010). Construct validity of the Chinese version of the Chalder Fatigue Scale in a Chinese community sample. J Psychosom Res.

[33] Tanaka M, Fukuda S, Mizuno K, Imai-Matsumura K, Jodoi T, Kawatani J, Takano M, Miike T, Tomoda A, Watanabe Y (2008). Reliability and validity of the Japanese version of the Chalder Fatigue Scale among youth in Japan. Psychol Rep.

[34] Becker P, Jansen LJ (2006). Chronischer Stress, Persönlichkeit und selbstberichtete körperliche Gesundheit. Z Gesundh.

[35] Tanaka JS, Bollen KAL JS (1993). Multifaceted Conceptions of Fit in Structural Equation Models. Testing Structural Equation Models.

[36] Cheung GWRR (2002). Evaluating goodness-of-fit-indexes for testing measurement invariance. Struct Equat Model.

[37] Rudolf M, Müller J (2004). Multivariate Verfahren.

[38] Winkler G, Stolzenberg H (1999). Der Sozialschichtindex im Bundesgesundheitssurvey. Das Gesundheitswesen.

[39] Hobfoll SE (1989). Conservation of resources. A new attempt at conceptualizing stress. Am Physiol.

[40] Holahan CJ, Moos RH, Holahan CK, Cronkite RC (1999). Resource loss, resource gain, and depressive symptoms: a 10-year model. J Pers Soc Psychol.

[41] Ellert U, Kurth BM (2013). [Health related quality of life in adults in Germany: results of the German Health Interview and Examination Survey for Adults (DEGS1)]. Bundesgesundheitsbla.

[42] Kocalevent RD, Hinz A, Brahler E, Klapp BF (2011). Determinants of fatigue and stress. BMC Res Notes.

[43] Kocalevent RD, Hinz A, Brahler E (2013). Standardization of a screening instrument (PHQ-15) for somatization syndromes in the general population. BMC Psychiatry.

[44] Kocalevent RD, Levenstein S, Fliege H, Schmid G, Hinz A, Brahler E, Klapp BF (2007). Contribution to the construct validity of the Perceived Stress Questionnaire from a population-based survey. J Psychosom Res.

[45] Mache S, Vitzthum K, Wanke E, David A, Klapp BF, Danzer G (2014). Exploring the impact of resilience, self-efficacy, optimism and organizational resources on work engagement. Work.

[46] Lampert T, Kroll L, Muters S, Stolzenberg H (2013). [Measurement of socioeconomic status in the German Health Interview and Examination Survey for Adults (DEGS1)]. Bundesgesundheitsbla.

[47] Lampert T, Kroll LE, von der Lippe E, Muters S, Stolzenberg H (2013). Socioeconomic status and health: results of the German Health Interview and Examination Survey for Adults (DEGS1). Bundesgesundheitsbla.

[48] Nazroo JY, Edwards AC, Brown GW (1997). Gender differences in the onset of depression following a shared life event: a study of couples. Psychol Med.

[49] Kessler RC, Mcleod JD (1984). Sex-differences in vulnerability to undesirable life events. Am Sociol Rev.

[50] Organisation WH (2003). Gender Disparities in Mental Health.

[CR51] The pre-publication history for this paper can be accessed here: http://www.biomedcentral.com/1471-2458/14/902/prepub

